# M-estimation in high-dimensional linear model

**DOI:** 10.1186/s13660-018-1819-3

**Published:** 2018-08-30

**Authors:** Kai Wang, Yanling Zhu

**Affiliations:** School of Statistics and Applied Mathematics, Anhui University of Finance and Economics, Bengbu, P.R. China

**Keywords:** 62F12, 62E15, 62J05, M-estimation, High-dimensionality, Variable selection, Oracle property, Penalized method

## Abstract

We mainly study the M-estimation method for the high-dimensional linear regression model and discuss the properties of the M-estimator when the penalty term is a local linear approximation. In fact, the M-estimation method is a framework which covers the methods of the least absolute deviation, the quantile regression, the least squares regression and the Huber regression. We show that the proposed estimator possesses the good properties by applying certain assumptions. In the part of the numerical simulation, we select the appropriate algorithm to show the good robustness of this method.

## Introduction

For the classical linear regression model $Y=X\beta+\varepsilon$, we are interested in the problem of variable selection and estimation, where $Y=(y_{1},y_{2},\ldots,y_{n})^{T}$ is the response vector, $X=(X_{1},X_{2},\ldots,X_{p_{n}})=(x_{1},x_{2},\ldots,x_{n})^{T}=(x_{ij})_{n\times p_{n}}$ is an $n\times p_{n}$ design matrix, and $\varepsilon=(\varepsilon_{1},\varepsilon_{2},\ldots,\varepsilon_{n})^{T}$ is a random vector. The main topic is how to estimate the coefficients vector $\beta\in\textrm{R}^{p}_{n}$ when $p_{n}$ increases with sample size *n* and many elements of *β* equal zero. We can transfer this problem into a minimization of a penalized least squares objective function

$$\hat{\beta_{n}}=\operatorname{arg}\min_{\beta}Q_{n}(\beta_{n}),\quad Q_{n}(\beta_{n})= \Vert Y-X\beta_{n} \Vert ^{2}+\sum _{j=1}^{p_{n}}p_{\lambda_{n}}\bigl( \vert \beta_{nj} \vert \bigr), $$

where $\Vert \cdot\Vert $ is the $l_{2}$ norm of the vector, $\lambda_{n}$ is a tuning parameter, and $p_{\lambda_{n}}(|t|)$ a penalty term. It is well known that the least squares estimation is not robust, especially when in the data there exist abnormal values or the error term has a heavy-tailed distribution.

In this paper we consider the loss function to be the least absolute deviation, i.e., we minimize the following objective function:

$$\hat{\beta_{n}}=\operatorname{arg}\min_{\beta}Q_{n}(\beta_{n}),\quad Q_{n}(\beta_{n})= \frac {1}{n}\sum_{i=1}^{n} \bigl\vert y_{i}-x_{i}^{T}\beta_{n} \bigr\vert +\sum_{j=1}^{p_{n}}p_{\lambda _{n}}\bigl( \vert \beta_{nj} \vert \bigr), $$

where the loss function is the least absolute deviation (LAD for short) that does not need the noise to obey a gaussian distribution and be more robust than a least squares estimation. In fact, the LAD estimation is a special case of the M-estimation, which was mentioned by Huber (1964, 1973, 1981) [1–3] firstly and which can be obtained by minimizing the objective function

$$Q_{n}(\beta_{n})=\frac{1}{n}\sum _{i=1}^{n}\rho\bigl(y_{i}-x_{i}^{T} \beta_{n}\bigr), $$

where the function *ρ* can be selected. For example, if we choose $\rho(x)=\frac{1}{2}x^{2}1_{|x|\leq c}+(c|x|-c^{2}/2)1_{|x|>c}$, where $c>0$, the Huber estimator can be obtained; if we choose $\rho(x)=|x|^{q}$, where $1\leq q\leq2$, $L_{q}$ estimator will be obtained, with two special cases: LAD estimator for $q=1$ and OLS estimator for $q=2$. If we choose $\rho(x)=\alpha x^{+}+(1-\alpha)(-x)^{+}$, where $0<\alpha<1$, $x^{+}=\max(x,0)$, we call it a quantile regression, and we can also get the LAD estimator for $\alpha=1/2$ especially.

When $p_{n}$ approaches infinity as *n* tends to infinity, we assume that the function *ρ* is convex and not monotone, and the monotone function *φ* is the derivative of *ρ*. By imposing the appropriate regularity conditions, Huber (1973), Portnoy (1984) [4], Welsh (1989) [5] and Mammen (1989) [6] have proved that the M-estimator enjoyed the properties of consistency and asymptotic normality, where Welsh (1989) gave the weaker condition imposed on *φ* and the stronger condition on $p_{n}/n$. Bai and Wu [7] further pointed out that the condition on $p_{n}$ could be a part of the integrable condition imposed on the design matrix. Moreover, He and Shao (2000) [8] studied the asymptotic properties of the M-estimator in the case of a generalized model setting and the dimension $p_{n}$ getting bigger and bigger. Li (2011) [9] obtained the Oracle property of the non-concave penalized M-estimator in high-dimensional model with the condition of $p_{n}\log n/n\rightarrow0$, $p_{n}^{2}/n\rightarrow0$, and proposed RSIS to make a variable selection by applying a rank sure independence screening method in the ultra high-dimensional model. Zou and Li (2008) [10] combined a penalized function and a local linear approximation method (LLA) to prove that the obtained estimator enjoyed good asymptotic properties, and they demonstrated that this method improved the computational efficiency of a local quadratic approximation (LQA) in a simulation.

Inspired by this, in this paper we consider the following problem:

1.1 $$ \hat{\beta_{n}}=\operatorname{arg}\min_{\beta_{n}} Q_{n}(\beta_{n}),\quad Q_{n}(\beta_{n})= \frac {1}{n}\sum_{i=1}^{n}\rho \bigl(y_{i}-x_{i}^{T}\beta_{n}\bigr)+ \sum_{j=1}^{p_{n}}p'_{\lambda _{n}} \bigl( \vert \tilde{\beta}_{nj} \vert \bigr) \vert \beta_{nj} \vert , $$

where $p'_{\lambda_{n}}(\cdot)$ is the derivative of the penalized function, and $\tilde{\beta}_{n}=(\tilde{\beta}_{n1},\tilde{\beta }_{n2},\ldots,\tilde{\beta}_{np_{n}})^{T}$ is the non-penalized estimator.

In this paper, we assume that the function *ρ* is convex, hence the objective function is still convex and the obtained local minimizer is a global minimizer.

## Main results

For convenience, we first give some notations. Let $\beta_{0}=(\beta_{01},\beta_{02},\ldots,\beta_{0p})^{T}$ be the true parameter. Without loss of generality, we assume the first $k_{n}$ coefficients of the covariates are nonzero, then there are $p_{n}-k_{n}$ covariates with zero coefficients. $\beta_{0}=(\beta_{0(1)}^{T}, \beta _{0(2)}^{T})^{T}$, $\hat{\beta_{n}}=(\hat{\beta}_{n(1)}^{T},\hat{\beta }_{n(2)}^{T})^{T}$ correspondingly. For the given symmetric matrix *Z*, denote by $\lambda_{\mathrm{min}}(Z)$ and $\lambda_{\mathrm{max}}(Z)$ the minimum and maximum eigenvalue of *Z*, respectively. Denote $\frac{X^{T}X}{n}:=D$ and $D=\left(\begin{array}{cc} D_{11} & D_{12}\\ D_{21} & D_{22} \end{array}\right)$, where $D_{11}=\frac{1}{n}X_{(1)}^{T}X_{(1)}$. Finally, we denote $c_{n}=\max \{|p'_{\lambda_{n}}(|\tilde{\beta}_{nj}|)|:\tilde{\beta}_{nj}\neq0,1\leq j\leq p_{n}\}$.

Next, we state some assumptions which will be needed in the following results.

$(A_{1})$
The function *ρ* is convex on *R*, and its left derivative and right derivative $\varphi_{+}(\cdot)$, $\varphi_{-}(\cdot)$ satisfies $\varphi_{-}(t)\leq\varphi(t)\leq\varphi_{+}(t)$, $\forall t\in R$.
$(A_{2})$
The error term *ε* is i.i.d, and the distribution function *F* of $\varepsilon_{i}$ satisfies $F(S)=0$, where *S* is the set of discontinuous points of *φ*.
  Moreover, $E[\varphi(\varepsilon_{i})]=0$, $0< E[\varphi^{2}(\varepsilon_{i})]=\sigma ^{2}<\infty$, and $G(t)\equiv E[\varphi(\varepsilon_{i}+t)]=\gamma t+o(|t|)$, where $\gamma >0$. Besides these, we assume that $\lim_{t\rightarrow0}E[\varphi(\varepsilon_{i}+t)-\varphi (\varepsilon_{i})]^{2}=0$.
$(A_{3})$
There exist constants $\tau_{1}$, $\tau_{2}$, $\tau_{3}$, $\tau _{4}$ such that $0<\tau_{1}\leq\lambda_{\mathrm{min}}(D)\leq\lambda _{\mathrm{max}}(D)\leq\tau_{2}$ and $0<\tau_{3}\leq\lambda_{\mathrm{min}}(D_{11})\leq\lambda_{\mathrm{max}}(D_{11})\leq\tau _{4}$.
$(A_{4})$
$\lambda_{n}\rightarrow0$ ($n\rightarrow\infty$), $p_{n}=O(n^{1/2})$, $c_{n}=O(n^{-1/2})$.
$(A_{5})$
Let $z_{i}$ be the transpose of the *i*th row vector of $X_{(1)}$, such that $\lim_{n\rightarrow\infty}n^{-\frac{1}{2}} \max_{1\leq i\leq n}z_{i}^{T}z_{i}=0$.

It is worth mentioning that conditions $(A_{1})$ and $(A_{2})$ are classical assumptions for an M-estimation in a linear model, which can be found in many references, for example Bai, Rao and Wu (1992) [11] and Wu (2007) [12]. The condition $(A_{3})$ is frequently used for a sparse model in the linear model regression theory, which requires that the eigenvalues of the matrices *D* and $D_{11}$ are bounded. The condition $(A_{4})$ is weaker than that in previous references. Under the condition $(A_{4})$ we broaden the order of $p_{n}$ to $n^{1/2}$, but Huber (1973) and Li, Peng and Zhu (2011) [9] required that $p_{n}^{2}/n\rightarrow0$, Portnoy (1984) required $p_{n}\log p_{n}/n\rightarrow0$, and Mammen (1989) required $p_{n}^{3/2}\log p_{n}/n\rightarrow0$. Compared with these results, it is obvious that our sparse condition is much weaker. The condition $(A_{5})$ is the same as that in Huang, Horowitz and Ma (2008) [13], which is used to prove the asymptotic properties of the nonzero part of M-estimation.

### Theorem 2.1

(Consistency of estimator)

*If the conditions* $(A_{1})$*–*$(A_{4})$ *hold*, *there exists a non*-*concave penalized M*-*estimation* $\hat{\beta_{n}}$, *such that*

$$\Vert \hat{\beta_{n}}-\beta_{0} \Vert =O_{P}\bigl((p_{n}/n)^{1/2}\bigr). $$

### Remark 2.1

From Theorem 2.1, we can see that there exists a global M-estimation $\hat{\beta_{n}}$ if we choose the appropriate tuning parameter $\lambda _{n}$; moreover, this M-estimation is $(n/p_{n})^{1/2}$-consistent. This convergence rate is the same as that in the work of Huber (1973) and Li, Peng and Zhu (2011).

### Theorem 2.2

(The sparse model)

*If the conditions* $(A_{1})$*–*$(A_{4})$ *hold and* $\lambda_{\mathrm{min}}(D)>\lambda_{\mathrm{max}}(\frac {1}{n} \sum_{i=1}^{n}J_{i}J_{i}^{T})$, *for the non*-*concave penalized M*-*estimation* $\hat{\beta_{n}}$ *we have*

$$P(\hat{\beta}_{n(2)}=0)\rightarrow1. $$

### Remark 2.2

By Theorem 2.2, we see that under the suitable conditions the global M-estimation of the zero-coefficient variables goes to zero with a high probability when *n* is large enough. This also shows that the model is sparse.

### Theorem 2.3

(Oracle property)

*If the conditions* $(A_{1})$*–*$(A_{5})$ *hold and* $\lambda_{\mathrm{min}}(D)>\lambda_{\mathrm{max}}(\frac {1}{n} \sum_{i=1}^{n}J_{i}J_{i}^{T})$, *with probability converging to one*, *the non*-*concave penalized M*-*estimation* $\hat{\beta_{n}}=(\hat{\beta}_{n(1)}^{T},\hat{\beta}_{n(2)}^{T})^{T}$ *has the following properties*:

1. (*The consistency of the model selection*) $\hat{\beta}_{n(2)}=0$;
2. (*Asymptotic normality*)
  $$\begin{aligned}[b]\sqrt{n}s_{n}^{-1}u^{T}( \hat{\beta}_{n(1)}-\beta _{0(1)})&=\sum _{i=1}^{n}n^{-1/2}s_{n}^{-1} \gamma^{-1}u^{T}D_{11}z_{i}^{T} \varphi (\varepsilon_{i})+o_{P}(1)\stackrel{\mathrm{d}}{ \longrightarrow}N(0,1), \end{aligned} $$
  *where* $s_{n}^{2}=\sigma^{2}\gamma^{-1}u^{T}D_{11}^{-1}u$, *and* *u* *is any* $k_{n}$ *dimensional vector such that* $\|u\|\leq1$. *Meanwhile*, $z_{i}$ *is the transpose of the* *ith row vector of a* $k_{n}\times k_{n}$ *matrix* $X_{(1)}$.

### Remark 2.3

From Theorem 2.3, the M-estimation enjoys the Oracle property, that is, the M-estimator can correctly select covariates with nonzero coefficients with probability converging to one and the estimators of the nonzero coefficients has the same asymptotic distribution that they would have if the zero coefficients were known in advance.

### Remark 2.4

In Fan and Peng (2004) [14], the authors showed that the non-concave penalized M-estimation has the property of consistency with the condition $p_{n}^{4}/n\rightarrow0$, and enjoyed the property of asymptotic normality with the condition $p_{n}^{5}/n\rightarrow0$. By Theorems 2.1–2.3, we can see that the corresponding conditions we impose are quite weak.

## Proofs of main results

### The proof of Theorem 2.1

Let $\alpha_{n}=(p_{n}/n)^{1/2}+p_{n}^{1/2}c_{n}$. For any $p_{n}$-dimensional vector *u* with $\|u\|=C$, we only need to prove that there exists a great enough positive constant *C* such that

$$\liminf_{n\rightarrow\infty}P\Bigl\{ \inf_{\|u\|=C}Q_{n}( \beta_{0}+\alpha_{n} u)>Q_{n}( \beta_{0})\Bigr\} \geq1-\varepsilon, $$

for any $\varepsilon>0$, that is, there at least exists a local minimizer $\hat{\beta_{n}}$ such that $\| \hat{\beta_{n}}-\beta_{0}\|=O_{P}(\alpha_{n})$ in the closed ball $\{\beta_{0}+\alpha_{n} u:\|u\|\leq C\}$.

Firstly, by the triangle inequality we get

$$ \begin{aligned}[b] &Q_{n}(\beta_{0}+\theta u)-Q_{n}(\beta_{0}) \\ &\quad =\frac{1}{n}\sum_{i=1}^{n}\bigl[\rho \bigl(y_{i}-x_{i}^{T}(\beta_{0}+ \alpha_{n} u)\bigr)-\rho \bigl(y_{i}-x_{i}^{T} \beta_{0}\bigr)\bigr]+\sum_{j=1}^{p_{n}}p'_{\lambda_{n}} \bigl( \vert \tilde{\beta }_{nj} \vert \bigr) \bigl( \vert \beta_{0j}+\alpha_{n} u_{j} \vert - \vert \beta_{0j} \vert \bigr) \\ &\quad \geq\frac{1}{n}\sum_{i=1}^{n}\bigl[ \rho\bigl(y_{i}-x_{i}^{T}(\beta_{0}+ \alpha_{n} u)\bigr)-\rho \bigl(y_{i}-x_{i}^{T} \beta_{0}\bigr)\bigr]-\alpha_{n}\sum _{j=1}^{p_{n}}p'_{\lambda_{n}}\bigl( \vert \tilde{\beta }_{nj} \vert \bigr) \vert u_{j} \vert \\ &\quad :=T_{1}+T_{2}, \end{aligned} $$

where $T_{1}=\frac{1}{n}\sum_{i=1}^{n}[\rho(y_{i}-x_{i}^{T}(\beta_{0}+\alpha_{n} u))-\rho(y_{i}-x_{i}^{T}\beta_{0})]$, $T_{2}=-\alpha_{n}\sum_{j=1}^{p_{n}}p'_{\lambda _{n}}(|\tilde{\beta}_{nj}|)|u_{j}|$. Noticing that

3.1 $$ \begin{aligned}[b] T_{1}&=\frac{1}{n}\sum _{i=1}^{n}\bigl[\rho\bigl(y_{i}-x_{i}^{T}( \beta _{0}+\alpha_{n} u)\bigr)-\rho\bigl(y_{i}-x_{i}^{T} \beta_{0}\bigr)\bigr] \\ &=\frac{1}{n}\sum_{i=1}^{n}\bigl[\rho \bigl(\varepsilon_{i}-\alpha_{n} x_{i}^{T}u \bigr)-\rho (\varepsilon_{i})\bigr] \\ &=\frac{1}{n}\sum_{i=1}^{n} \int_{0}^{-\alpha_{n} x_{i}^{T}u}\bigl[\varphi(\varepsilon _{i}+t)-\varphi(\varepsilon_{i})\bigr]\,dt-\frac{1}{n} \alpha_{n} \sum_{i=1}^{n}\varphi( \varepsilon_{i})x_{i}^{T}u \\ &:=T_{11}+T_{12}, \end{aligned} $$

where $T_{11}=\frac{1}{n}\sum_{i=1}^{n}\int_{0}^{-\alpha_{n} x_{i}^{T}u}[\varphi (\varepsilon_{i}+t)-\varphi(\varepsilon_{i})]\,dt$, $T_{12}=-\frac{1}{n}\alpha _{n} \sum_{i=1}^{n}\varphi(\varepsilon_{i})x_{i}^{T}u$. Combining with the Von-Bahr–Esseen inequality and the fact that $|T_{12}|\leq\frac{1}{n}\alpha_{n} \|u\|\|\sum_{i=1}^{n}\varphi (\varepsilon_{i})x_{i}\|$, we instantly have

$$E\Biggl[ \Biggl\Vert \sum_{i=1}^{n} \varphi(\varepsilon_{i})x_{i} \Biggr\Vert ^{2} \Biggr] \leq n\sum_{i=1}^{n}E\bigl[ \bigl\Vert \varphi(\varepsilon_{i})x_{i} \bigr\Vert ^{2}\bigr]=n\sum_{i=1}^{n}E[ \varphi^{2}(\varepsilon_{i})x_{i}^{T}x_{i} \leq n^{2}p_{n}\sigma^{2}, $$

hence

3.2 $$ \vert T_{12} \vert =O_{P}\bigl(\alpha_{n}p_{n}^{1/2} \bigr) \Vert u \Vert =O_{P}\bigl(\bigl(p_{n}^{2}/n \bigr)^{1/2}\bigr). $$

Secondly for $T_{11}$, let $T_{11}=\sum_{i=1}^{n}A_{in}$, where $A_{in}=\frac{1}{n}\int_{0}^{-\alpha_{n} x_{i}^{T}u}[\varphi(\varepsilon _{i}+t)-\varphi(\varepsilon_{i})]\,dt$, so

$$T_{11}=\sum_{i=1}^{n} \bigl[A_{in}-E(A_{in})\bigr]+\sum _{i=1}^{n}E(A_{in}):=T_{111}+T_{112}. $$

We can easily obtain $E(T_{111})=0$. From the Von-Bahr–Esseen inequality, the Schwarz inequality and the condition $(B_{3})$, it follows that

$$\begin{aligned}[b] \operatorname{var}(T_{111})&=\operatorname{var}\Biggl(\sum _{i=1}^{n}A_{in}\Biggr)\leq\frac {1}{n} \sum_{i=1}^{n}E \biggl( \int_{0}^{-\alpha_{n} x_{i}^{T}u}\bigl[\varphi (\varepsilon_{i}+t)- \varphi(\varepsilon_{i})\bigr]\,dt \biggr)^{2} \\ &\leq\frac{1}{n}\sum_{i=1}^{n} \bigl\vert \alpha_{n} x_{i}^{T}u \bigr\vert \biggl\vert \int_{0}^{-\alpha_{n} x_{i}^{T}u}E\bigl[\varphi(\varepsilon_{i}+t)- \varphi(\varepsilon_{i})\bigr]^{2}\,dt \biggr\vert \\ &=\frac{1}{n}\sum_{i=1}^{n}o_{P}(1) \bigl(\alpha_{n} x_{i}^{T}u\bigr)^{2}= \frac {1}{n}o_{P}(1)\alpha_{n}^{2}\sum _{i=1}^{n}u^{T}x_{i}x_{i}^{T}u \\ &=o_{P}(1)\alpha_{n}^{2}u^{T}Du\leq \lambda_{\mathrm{max}}(D)o_{P}(1)\alpha_{n}^{2} \Vert u \Vert ^{2}=o_{P}\bigl(\alpha_{n}^{2} \bigr) \Vert u \Vert ^{2}, \end{aligned} $$

so together with the Markov inequality this yields

$$P\bigl( \vert T_{111} \vert >C_{1}\alpha_{n} \Vert u \Vert \bigr)\leq\frac{\operatorname{var}(T_{111})}{C_{1}^{2}\alpha_{n}^{2} \Vert u \Vert ^{2}}\leq\frac{o_{P}(\alpha_{n}^{2}) \Vert u \Vert ^{2}}{C_{1}^{2}\alpha_{n}^{2} \Vert u \Vert ^{2}}\rightarrow0\quad (n \rightarrow\infty), $$

hence

3.3 $$ T_{111}=o_{P}(\alpha_{n}) \Vert u \Vert . $$

As for $T_{112}$,

3.4 $$ \begin{aligned}[b] T_{112}&=\sum_{i=1}^{n}E(A_{in})= \frac{1}{n}\sum_{i=1}^{n} \int_{0}^{-\alpha_{n} x_{i}^{T}u}\bigl[\gamma t+o\bigl( \vert t \vert \bigr)\bigr]\,dt \\ &=\frac{1}{n}\sum_{i=1}^{n}\biggl( \frac{1}{2}\gamma\alpha _{n}^{2}u^{T}x_{i}x_{i}^{T}u+o_{P}(1) \alpha_{n}^{2}u^{T}x_{i}x_{i}^{T}u \biggr) \\ &=\frac{1}{2}\gamma\alpha_{n}^{2}u^{T}Du+o_{p}(1) \alpha_{n}^{2}u^{T}Du \\ &\geq\biggl[\frac{1}{2}\gamma\lambda_{\mathrm{min}}(D)+o_{P}(1) \biggr]\alpha_{n}^{2} \Vert u \Vert ^{2}. \end{aligned} $$

Finally, considering $T_{2}$, we can easily obtain

3.5 $$ T_{2}\leq(p_{n})^{1/2}\alpha_{n}\max \bigl\{ \bigl\vert p'_{\lambda_{n}}\bigl( \vert \tilde{\beta }_{nj} \vert \bigr) \bigr\vert ,1\leq j\leq k_{n}\bigr\} \Vert u \Vert =(p_{n})^{1/2}\alpha_{n}c_{n} \Vert u \Vert \leq\alpha_{n}^{2} \Vert u \Vert . $$

This together with (3.1)–(3.5) shows that we can choose a great enough constant *C* such that $T_{111}$ and $T_{2}$ are controlled by $T_{112}$, from which it follows that there at least exists a local minimizer $\hat {\beta_{n}}$ such that $\|\hat{\beta_{n}}-\beta_{0}\|=O_{P}(\alpha_{n})$ in the closed ball $\{\beta_{0}+\alpha_{n} u:\|u\|\leq C\}$. □

### The proof of Theorem 2.2

From Theorem 2.1, as long as we choose a great enough constant *C* and appropriate $\alpha_{n}$, then $\hat{\beta_{n}}$ will be in the ball $\{ \beta_{0}+\alpha_{n} u:\|u\|\leq C\}$ with probability converging to one, where $\alpha_{n}=(p_{n}/n)^{1/2}+p_{n}^{1/2}c_{n}$. For any $p_{n}$-dimensional vector $\beta_{n}$, now we denote $\beta_{n}=(\beta_{n(1)}^{T},\beta_{n(2)}^{T})^{T}$, $\beta_{n(1)}=\beta _{0(1)}+\alpha_{n} u_{(1)}$, $\beta_{n(2)}=\beta_{0(2)}+\alpha_{n} u_{(2)}=\alpha_{n} u_{(2)}$, where $\beta_{0}=(\beta_{0(1)}^{T},\beta _{0(2)}^{T})^{T}$, $\|u\|^{2}=\|u_{(1)}\|^{2}+\|u_{(2)}\|^{2}\leq C^{2}$. Meanwhile let

$$V_{n}(u_{(1)},u_{(2)})=Q_{n}\bigl(\bigl( \beta_{n(1)}^{T},\beta_{n(2)}^{T} \bigr)^{T}\bigr)-Q_{n}\bigl(\bigl(\beta _{0(1)}^{T},0^{T} \bigr)^{T}\bigr), $$

then by minimizing $V_{n}(u_{(1)},u_{(2)})$ we can obtain the estimator $\hat{\beta_{n}}=(\hat{\beta}_{n(1)}^{T},\hat{\beta}_{n(2)}^{T})^{T}$, where $\| u_{(1)}\|^{2}+\|u_{(2)}\|^{2}\leq C^{2}$. In the following part, we will prove that, as long as $\|u\|\leq C$, $\| u_{(2)}\|>0$,

$$P\bigl(V_{n}(u_{(1)},u_{(2)})-V_{n}(u_{(1)},0)>0 \bigr)\rightarrow1\quad (n\rightarrow\infty) $$

holds, for any $p_{n}$-dimensional vector $u=(u_{(1)}^{T},u_{(2)}^{T})^{T}$. We can easily find the fact that

$$\begin{aligned} &V_{n}(u_{(1)},u_{(2)})-V_{n}(u_{(1)},0)\\ &\quad =Q_{n}\bigl(\bigl(\beta_{n(1)}^{T}, \beta_{n(2)}^{T}\bigr)^{T}\bigr)-Q_{n} \bigl(\bigl(\beta_{n(1)}^{T},0^{T}\bigr)^{T} \bigr) \\ &\quad =\frac{1}{n}\sum_{i=1}^{n}\bigl[\rho \bigl(\varepsilon_{i}-\alpha_{n} H_{i}^{T}u_{(1)}- \alpha_{n} J_{i}^{T}u_{(2)}\bigr)-\rho \bigl(\varepsilon_{i}-\alpha_{n} H_{i}^{T}u_{(1)} \bigr)\bigr] +\sum_{j=k_{n}+1}^{p_{n}}p'_{\lambda_{n}} \bigl( \vert \tilde{\beta}_{nj} \vert \bigr) \vert \alpha_{n} u_{j} \vert \\ &\quad =\frac{1}{n}\sum_{i=1}^{n} \int_{-\alpha_{n} H_{i}^{T}u_{(1)}}^{-\alpha_{n} H_{i}^{T}u_{(1)}-\alpha_{n} J_{i}^{T}u_{(2)}} \bigl[\varphi(\varepsilon_{i}+t)- \varphi(\varepsilon_{i})\bigr]\,dt-\frac{1}{n}\alpha _{n}\sum_{i=1}^{n}\varphi( \varepsilon_{i})J_{i}^{T}u_{(2)} \\ &\qquad {} +\sum_{j=k_{n}+1}^{p_{n}}p'_{\lambda_{n}} \bigl( \vert \tilde{\beta}_{nj} \vert \bigr) \vert \alpha_{n} u_{j} \vert \\ &\quad :=W_{1}+W_{2}+W_{3}, \end{aligned}$$

where $H_{i}$ and $J_{i}$ are $k_{n}$ and $p_{n}-k_{n}$ dimensional vectors, respectively, such that $x_{i}=(H_{i}^{T}+J_{i}^{T})^{T}$. Similar to the proof of Theorem 2.1, we get

3.6 $$\begin{aligned}& \begin{aligned}[b] W_{1}&=\frac{1}{n}\sum _{i=1}^{n} \int_{-\alpha_{n} H_{i}^{T}u_{(1)}}^{-\alpha_{n} H_{i}^{T}u_{(1)}-\alpha_{n} J_{i}^{T}u_{(2)}} \bigl[\varphi(\varepsilon_{i}+t)- \varphi(\varepsilon_{i})\bigr]\,dt \\ &=\frac{1}{2n}\sum_{i=1}^{n}\gamma \alpha_{n}^{2}u^{T}x_{i}x_{i}^{T}u- \frac {1}{2n}\sum_{i=1}^{n}\gamma \alpha_{n}^{2}u_{(2)}^{T}J_{i}J_{i}^{T}u_{(2)} +o_{P}(1)\alpha_{n}^{2} \Vert u \Vert ^{2}+o_{P}(1)\alpha_{n} \Vert u \Vert \\ &\geq\frac{1}{2}\gamma\alpha_{n}^{2}\Biggl[ \lambda_{\mathrm{min}}(D)-\lambda_{\mathrm{max}}\Biggl(\frac {1}{n}\sum _{i=1}^{n}J_{i}J_{i}^{T} \Biggr)\Biggr] \Vert u \Vert ^{2} +o_{P}(1) \alpha_{n}^{2} \Vert u \Vert ^{2}+o_{P}(1) \alpha_{n} \Vert u \Vert , \end{aligned} \end{aligned}$$

3.7 $$\begin{aligned}& \vert W_{2} \vert = \Biggl\vert -\frac{1}{n} \alpha_{n}\sum_{i=1}^{n}\varphi( \varepsilon_{i})J_{i}^{T}u_{(2)} \Biggr\vert =O_{P}\bigl(\bigl(p_{n}^{2}/n \bigr)^{1/2}\bigr) \Vert u \Vert , \end{aligned}$$

and

3.8 $$ \begin{aligned}[b] \vert W_{3} \vert &= \Biggl\vert \sum_{j=k_{n}+1}^{p_{n}}p'_{\lambda_{n}} \bigl( \vert \tilde{\beta }_{nj} \vert \bigr) \vert \alpha_{n} u_{j} \vert \Biggr\vert \leq(p_{n})^{1/2} \alpha_{n}\max\bigl\{ \bigl\vert p'_{\lambda_{n}}\bigl( \vert \tilde{\beta }_{nj} \vert \bigr) \bigr\vert ,k_{n}+1\leq j\leq p_{n}\bigr\} \Vert u \Vert \\ &=(p_{n})^{1/2}\alpha_{n}c_{n} \Vert u \Vert \leq\alpha_{n}^{2} \Vert u \Vert . \end{aligned} $$

By Eqs. (3.6)–(3.8) and the condition $\lambda_{\mathrm{min}}(D)>\lambda _{\mathrm{max}}(\frac{1}{n}\sum_{i=1}^{n}J_{i}J_{i}^{T})$, it follows that

$$\begin{aligned}[b] &V_{n}(u_{(1)},u_{(2)})-V_{n}(u_{(1)},0) \\ &\quad \geq\frac{1}{2}\gamma\alpha_{n}^{2}\Biggl[ \lambda_{\mathrm{min}}(D)-\lambda_{\mathrm{max}}\Biggl(\frac {1}{n}\sum _{i=1}^{n}J_{i}J_{i}^{T} \Biggr)\Biggr] \Vert u \Vert ^{2} \\ &\qquad {} +o_{P}(1)\alpha_{n}^{2} \Vert u \Vert ^{2}+o_{P}(1)\alpha_{n} \Vert u \Vert +O_{P}\bigl(\bigl(p_{n}^{2}/n \bigr)^{1/2}\bigr) \Vert u \Vert +O_{P}\bigl( \alpha_{n}^{2}\bigr) \Vert u \Vert \\ &\quad >0, \end{aligned} $$

which shows that, as long as $\|u\|\leq C$, $\|u_{(2)}\|>0$,

$$P\bigl(V_{n}(u_{(1)},u_{(2)})-V_{n}(u_{(1)},0)>0 \bigr)\rightarrow1\quad (n\rightarrow\infty) $$

holds, for any $p_{n}$-dimensional vector $u=(u_{(1)}^{T},u_{(2)}^{T})^{T}$. □

### The proof of Theorem 2.3

It is obvious that the conclusion (1) can be obtained instantly by Theorem 2.2, so we only need to prove the conclusion (2). It follows from Theorem 2.1 that $\hat{\beta_{n}}$ is consistent with $\beta_{0}$ and $\hat{\beta}_{n(2)}=0$ with probability converging to one from Theorem 2.2. Therefore for $\hat{\beta}_{n(1)}$

$$\frac{\partial Q_{n}(\beta_{n})}{\partial\beta_{n(1)}}\bigg|_{\beta _{n(1)}=\hat{\beta}_{n(1)}}=0, $$

that is,

$$-\frac{1}{n}\sum_{i=1}^{n}H_{i} \varphi\bigl(y_{i}-H_{i}^{T}\hat{\beta }_{n(1)}\bigr)+W_{(1)}=0, $$

where

$$W=\bigl(p'_{\lambda_{n}}\bigl( \vert \tilde{ \beta}_{n1} \vert \bigr)\operatorname{sgn}(\hat{\beta}_{n1}),p'_{\lambda _{n}} \bigl( \vert \tilde{\beta}_{n2} \vert \bigr)\operatorname{sgn}(\hat{ \beta}_{n2}), \ldots,p'_{\lambda_{n}}\bigl( \vert \tilde{\beta}_{np_{n}} \vert \bigr)\operatorname{sgn}(\hat{\beta}_{np_{n}}) \bigr)^{T}. $$

In the following part we give the Taylor expansion of upper left first term:

$$\begin{aligned}[b] -\frac{1}{n}\sum_{i=1}^{n} \bigl\{ H_{i}\varphi\bigl(y_{i}-H_{i}^{T} \hat {\beta}_{0(1)}\bigr)- \bigl[\varphi' \bigl(y_{i}-H_{i}^{T}\beta_{0(1)} \bigr)H_{i}H_{i}^{T}+o_{P}(1)\bigr]( \hat{\beta }_{n(1)}-\beta_{0(1)})\bigr\} +W_{(1)}=0. \end{aligned} $$

Noticing that $y_{i}=H_{i}^{T}\beta_{0(1)}+\varepsilon_{i}$, we have

$$\begin{aligned}[b] -\frac{1}{n}\sum_{i=1}^{n}H_{i} \varphi(\varepsilon _{i})+\frac{1}{n}\sum _{i=1}^{n}\bigl[\varphi'( \varepsilon_{i})H_{i}H_{i}^{T}+o_{P}(1) \bigr] (\hat{\beta}_{n(1)}-\beta_{0(1)})+W_{(1)}=0, \end{aligned} $$

which shows that

$$\begin{aligned}[b] \frac{1}{n}\gamma\sum _{i=1}^{n}H_{i}H_{i}^{T}( \hat{\beta }_{n(1)}-\beta_{0(1)}) &=\frac{1}{n}\sum _{i=1}^{n}H_{i}\varphi( \varepsilon_{i})-W_{(1)}+(\hat{\beta }_{n(1)}- \beta_{0(1)})o_{P}(1) \\ &\quad {}+\frac{1}{n}\sum_{i=1}^{n}\bigl( \gamma-\varphi'(\varepsilon_{i})\bigr)H_{i}H_{i}^{T}( \hat {\beta}_{n(1)}-\beta_{0(1)}). \end{aligned} $$

Then, as long as $\|u\|\leq1$,

$$\begin{aligned}[b] u^{T}(\hat{\beta}_{n(1)}- \beta_{0(1)}) &=n^{-1}\gamma^{-1}u^{T}D_{11}^{-1} \sum_{i=1}^{n}H_{i}\varphi( \varepsilon _{i}) \\ &\quad {}+n^{-1}\gamma^{-1}u^{T}D_{11}^{-1} \sum_{i=1}^{n}\bigl(\gamma-\varphi '(\varepsilon_{i})\bigr)H_{i}H_{i}^{T}( \hat{\beta}_{n(1)}-\beta_{0(1)}) \\ &\quad {}-\gamma^{-1}u^{T}D_{11}^{-1}W_{(1)}+o_{P}( \alpha_{n}) \end{aligned} $$

holds, for any $k_{n}$-dimensional vector *u*. For the upper right third term, we can obtain

3.9 $$ \begin{aligned}[b] \bigl\vert \gamma^{-1}u^{T}D_{11}^{-1}W_{(1)} \bigr\vert &\leq\frac{1}{\gamma \lambda_{\mathrm{min}}(D_{11})} \Vert W_{(1)} \Vert \leq \frac{1}{\gamma\lambda_{\mathrm{min}}(D_{11})}p_{n}^{1/2}c_{n}\\ & \leq \frac{\alpha_{n}}{\gamma\lambda_{\mathrm{min}}(D_{11})}\rightarrow o_{P}(1)\quad (n\rightarrow\infty) . \end{aligned} $$

Now let us deal with the upper right second term. Theorem 2.1 and the condition $(A_{3})$ yield

3.10 $$ \begin{aligned}[b] & \Biggl\vert n^{-1}\gamma^{-1}u^{T}D_{11}^{-1} \sum_{i=1}^{n}\bigl(\gamma - \varphi'(\varepsilon_{i})\bigr)H_{i}H_{i}^{T}( \hat{\beta}_{n(1)}-\beta_{0(1)}) \Biggr\vert \\ &\quad \leq\frac{1}{n\gamma\lambda_{\mathrm{min}}(D_{11})} \Biggl\Vert \sum_{i=1}^{n} \bigl(\gamma -\varphi'(\varepsilon_{i}) \bigr)H_{i}H_{i}^{T}(\hat{\beta}_{n(1)}- \beta_{0(1)}) \Biggr\Vert \\ &\quad \leq\frac{1}{n\gamma\lambda_{\mathrm{min}}(D_{11})} \Biggl\Vert \sum_{i=1}^{n} \bigl(\gamma -\varphi'(\varepsilon_{i}) \bigr)H_{i}H_{i}^{T} \Biggr\Vert \Vert \hat{ \beta}_{n(1)}-\beta_{0(1)} \Vert \\ &\quad \leq\frac{O_{P}(1)}{n\gamma\lambda_{\mathrm{min}}(D_{11})} \Vert \hat{\beta }_{n(1)}-\beta_{0(1)} \Vert =O_{P}\bigl(p_{n}^{1/2}n^{-3/2} \bigr), \end{aligned} $$

where the upper third inequality sign holds because of Lemma 3 of Mammen (1989). Combining (3.9)–(3.10), we have

$$\begin{aligned}[b] u^{T}(\hat{\beta}_{n(1)}- \beta_{0(1)}) =n^{-1}\gamma^{-1}u^{T}D_{11}^{-1} \sum_{i=1}^{n}H_{i}\varphi( \varepsilon _{i})+O_{P}(\alpha_{n})+O_{P} \bigl(p_{n}^{1/2}n^{-3/2}\bigr), \end{aligned} $$

that is,

$$n^{1/2}u^{T}(\hat{\beta}_{n(1)}-\beta_{0(1)})=n^{-1/2} \gamma ^{-1}u^{T}D_{11}^{-1}\sum _{i=1}^{n}H_{i}\varphi( \varepsilon_{i})+o_{P}(1).$$

Denote $s_{n}^{2}=\sigma^{2}\gamma^{-1}u^{T}D_{11}^{-1}u$, $F_{in}=n^{-1/2}s_{n}^{-1}\gamma^{-1}u^{T}D_{11}^{-1}z_{i}^{T}$, where $z_{i}$ is a $k_{n}\times k_{n}$ matrix and the transpose of the *i*th row vector of $X_{(1)}$, then $n^{1/2}u^{T}(\hat{\beta}_{n(1)}-\beta_{0(1)})=\sum_{i=1}^{n}F_{in}\varphi (\varepsilon_{i})+o_{P}(1)$. It follows from $(A_{5})$ that

$$\begin{aligned}[b] \sum_{i=1}^{n}F^{2}_{in}&= \sum_{i=1}^{n}F_{in}F'_{in} =\sum_{i=1}^{n}\bigl(n^{-1/2}s_{n}^{-1} \gamma ^{-1}u^{T}D_{11}^{-1}z_{i}^{T} \bigr) \bigl(n^{-1/2}s_{n}^{-1}\gamma^{-1}z_{i}D_{11}^{-1}u \bigr) \\ &=\sum_{i=1}^{n}n^{-1}s_{n}^{-2} \gamma^{-2}u^{T}D_{11}^{-1}z_{i}^{T}z_{i}D_{11}^{-1}u =s_{n}^{-2}\gamma^{-2}u^{T}D_{11}^{-1}u= \sigma^{-2}. \end{aligned} $$

Applying the Slutsky theorem, we see that

$$\sqrt{n}s_{n}^{-1}u^{T}(\hat{ \beta}_{n(1)}-\beta_{0(1)})\stackrel{\mathrm {d}}{ \longrightarrow}N(0,1). $$

 □

## Simulation results

In this section we evaluate the performance of the M-estimator proposed in (1.1) by simulation studies.

We begin with the data. Simulating the data by the model $Y=X\beta +\varepsilon$, where $\beta_{0(1)}=(-2,2.5,3,-1)^{T}$, *ε* follows $N(0,1)$, $t_{5}$ and mixed normally distribution $0.9N(0,1)+ [4]0.1N(0,9)$, respectively. The design matrix *X* is generated by a p-dimensional multivariate normal distribution with mean zero and covariance matrix whose $(i,j)$th component is $\rho^{|i-j|}$, where we set $\rho=0.5$.

Then for the loss function. In this section we can choose some special loss functions, such as the LAD loss function, the OLS loss function and the Huber loss function. In this paper we choose the LAD loss function and the Huber loss function.

About the penalty function: for $p'_{\lambda_{n}}(|\widetilde{\beta }_{nj}|)$ in the penalty function, we choose the penalty function as a SACD estimation in the following:

$$p_{\lambda_{n}}\bigl( \vert \beta \vert \bigr)= \textstyle\begin{cases} \lambda_{n} \vert \beta \vert , & 0\leq \vert \beta \vert \leq\lambda_{n}, \\ -(\beta^{2}-2a\lambda_{n} \vert \beta \vert +\lambda_{n}^{2})/(2(a-1)), & \lambda_{n}< \vert \beta \vert < a\lambda_{n}, \\ (a+1)\lambda_{n}^{2}/2, & \vert \beta \vert > a\lambda_{n}, \end{cases} $$

then $p'_{\lambda_{n}}(|\widetilde{\beta}_{nj}|)= \lambda_{n}I(|\widetilde{\beta}_{nj}|\leq\lambda_{n})+\frac{a\lambda _{n}-|\widetilde{\beta}_{nj}|}{a-1}I(\lambda_{n}<|\widetilde{\beta }_{nj}|\leq a\lambda_{n})$. By the proposal of Fan and Li (2001), we can select $a=3.7$, which shows that generalized cross validation can be applied in searching the best tuning parameter $\lambda_{n}$.

About stimulation algorithm. For the proposed LLA method, we connect the penalty function with independent variables and an independent variable, respectively, then we write a program by using the quantile package in R. For the Lasso method, we use the Lars package to simulate.

Now we address the selection of the tuning parameter. We apply the BIC criterion to select the tuning parameter. The criterion is

$$\operatorname{BIC}(\lambda_{n})=\ln\Biggl(\frac{1}{n}\sum _{i=1}^{n}\rho\bigl(y_{i}-x_{i}^{T} \hat{\beta }\bigr)\Biggr)+\mathit{DF}_{\lambda_{n}}\ln(n)/n, $$

where $\mathit{DF}_{\lambda_{n}}$ is the generalized degree of freedom used by Fan and Li (2001).

About the selection of the evaluation index. In order to evaluate the performance of the estimators, we select four measures called EE, PE, C, IC and CP, which are obtained by 500 replicates. EE is the median of $\Vert \hat{\beta}-\beta_{0}\Vert _{2}$ to evaluate the estimation accuracy, and PE is the prediction error defined by the median of $n^{-1}\|Y-X\hat{\beta }\|^{2}$. The other three measures are to qualify the performance of model consistency, where C and IC refer to the average number of correctly selected zero covariates and the average number of incorrectly selected zero covariates, and CP is the proportion of the number of the correct selection of zero variables to the total number of zero variables.

In the following we will compare the performances of the method LLA we proposed, the Lasso method and the Oracle estimation. Set $n=200,500,700$, respectively, and $p=[2\sqrt{n}]$.

From Table 1, we notice that the indices EE, C, IC, CP of our proposed LLA method perform better when $\varepsilon\sim N(0,1)$. In particular, for the index CP, LLA outperforms Lasso. The reason for this may be that we impose different penalties for important and unimportant variables, while Lasso imposes the same penalties for all variables. Moreover, with the increase of the sample size, the ability of LLA method to correctly identify unimportant variables is also increasing. When the sample size is 700 and the number of explanatory variables is 53, an average of 48.9617 unimportant variables-zero variables are estimated to be zero on average, with an average accuracy of 99.92%.

**Table 1:** Simulation results for LAD loss function and $\varepsilon\sim N(0,1)$

Setting	Method	EE	PE	C	IC	CP
*n* = 200	Oracle	10.8544	3.3916	24.0000	0	100%
*p* = 28	Lasso	10.5726	3.3035	10.8480	0	45.20%
*m* = 24	LLA	10.9153	3.3947	23.8540	0	99.39%
*n* = 500	Oracle	19.9085	5.4118	41.0000	0	100%
*p* = 45	Lasso	19.5952	5.2928	18.9920	0	46.32%
*m* = 41	LLA	19.9233	5.4045	40.9140	0	99.79%
*n* = 700	Oracle	24.3006	6.3847	49.0000	0	100%
*p* = 53	Lasso	24.0315	6.2994	23.1009	0	47.14%
*m* = 49	LLA	24.3666	6.4077	48.9617	0	99.92%

An interesting fact can be found from Table 2, that is, when the error term is chosen as $t_{5}$, the accuracy of the method LLA proposed to correctly exclude incorrect variables is slightly higher than that of the case where the error term is a standardized normal distribution. The reason is that when the error term is heavy tailed, it is more appropriate to choose LLA, but the accuracy of estimation and prediction is slightly worse than that of Lasso. When the sample size increases, the LLA and Oracle estimates perform equally well in the selection of important variables and the complexity of the model.

**Table 2:** Simulation results for LAD loss function and $\varepsilon\sim t_{5}$

Setting	Method	EE	PE	C	IC	CP
*n* = 200	Oracle	10.5634	4.2892	24.0000	0	100%
*p* = 28	Lasso	10.2810	4.1649	11.7700	0	49.04%
*m* = 24	LLA	10.6448	4.2725	23.8780	0	99.49%
*n* = 500	Oracle	19.4296	6.8240	41.0000	0	100%
*p* = 45	Lasso	19.1157	6.7042	18.9580	0	46.24%
*m* = 41	LLA	19.4665	6.8335	40.9560	0	99.89%
*n* = 700	Oracle	23.7784	8.0637	49.0000	0	100%
*p* = 53	Lasso	23.4389	7.9551	22.8800	0	46.69%
*m* = 49	LLA	23.7808	8.0919	48.9740	0	99.94%

As can be seen from Table 3, when the error term is set to a mixed normal distribution, the ability of the proposed method to correctly select zero variables is good. In the case of a small sample size, the ability of the Lasso method to select important variables is better.

**Table 3:** Simulation results for LAD loss function and $\varepsilon\sim 0.9N(0,1)+0.1N(0,9)$

Setting	Method	EE	PE	C	IC	CP
*n* = 200	Oracle	10.4815	4.4830	24.0000	0	100%
*p* = 28	Lasso	10.2030	4.4063	11.6360	0	48.48%
*m* = 24	LLA	10.5826	4.4529	23.9240	0	99.68%
*n* = 500	Oracle	19.2539	7.1997	41.0000	0	100%
*p* = 45	Lasso	18.9670	7.0960	19.3840	0	47.28%
*m* = 41	LLA	19.2950	7.1173	40.9520	0	99.88%
*n* = 700	Oracle	23.6354	8.5657	49.0000	0	100%
*p* = 53	Lasso	23.2424	8.4609	23.0580	0	47.06%
*m* = 49	LLA	23.6566	8.3699	48.9300	0	99.86%

From Tables 4–6 where we choose the Huber loss function, the LLA method we proposed behaves well both in variable selection and robustness. Compared with Table 1 and Table 4, when the data has outliers, we should choose LAD as the loss function. Moreover, when the error term follows a mixed normally distribution, the LLA method behaves better than the Lasso method. The reason for this is that the real data has a mixed normal distribution with high probability.

**Table 4:** Simulation results for Huber loss function and $\varepsilon \sim N(0,1)$

Setting	Method	EE	PE	C	IC	CP
*n* = 200	Oracle	10.8300	3.3696	24.0000	0	100%
*p* = 28	Lasso	9.6422	3.5569	20.0920	0	83.72%
*m* = 24	LLA	10.9088	3.3784	22.7200	0	94.67%
*n* = 500	Oracle	19.9141	5.4034	41.0000	0	100%
*p* = 45	Lasso	18.0691	5.6068	38.0300	0	92.76%
*m* = 41	LLA	19.8884	5.3937	40.5160	0	98.82%
*n* = 700	Oracle	24.3265	6.3761	49.0000	0	100%
*p* = 53	Lasso	22.4030	6.5988	46.2440	0	94.38%
*m* = 49	LLA	24.3596	6.3882	48.6620	0	99.31%

**Table 5:** Simulation results for Huber loss function and $\varepsilon \sim t_{5}$

Setting	Method	EE	PE	C	IC	CP
*n* = 200	Oracle	10.5572	4.2666	24.0000	0	100%
*p* = 28	Lasso	9.2590	4.4065	18.4680	0.0020	76.95%
*m* = 24	LLA	10.6099	4.2429	22.8100	0	95.04%
*n* = 500	Oracle	19.4395	6.8118	41.0000	0	100%
*p* = 45	Lasso	17.4385	6.9993	36.2080	0	88.31%
*m* = 41	LLA	19.4471	6.8247	40.5440	0	98.89%
*n* = 700	Oracle	23.8089	8.0487	49.0000	0	100%
*p* = 53	Lasso	21.6534	8.2558	44.4980	0	90.81%
*m* = 49	LLA	23.8220	8.0807	48.6940	0	99.38%

**Table 6:** Simulation results for Huber loss function and $\varepsilon \sim0.9N(0,1)+0.1N(0,9)$

Setting	Method	EE	PE	C	IC	CP
*n* = 200	Oracle	10.4829	4.4694	24.0000	0	100%
*p* = 28	Lasso	9.1630	4.6333	18.0680	0	75.28%
*m* = 24	LLA	10.5706	4.4827	22.7880	0	94.95%
*n* = 500	Oracle	19.2618	7.1860	41.0000	0	100%
*p* = 45	Lasso	17.3780	7.3190	35.3500	0	86.22%
*m* = 41	LLA	19.2962	7.2029	40.5900	0	99.00%
*n* = 700	Oracle	23.6356	8.5563	49.0000	0	100%
*p* = 53	Lasso	21.5202	8.7148	43.4420	0	88.66%
*m* = 49	LLA	23.6275	8.5822	48.7120	0	99.41%

## Conclusion

In this paper, we mainly study the M-estimation method for the high-dimensional linear regression model and discuss the properties of the M-estimator when the penalty term is the local linear approximation. We show that the proposed estimator possesses the good properties by applying certain assumptions. In the numerical simulation, we select the appropriate algorithm to show the good robustness of this method.
